# Supplementary material for: Simultaneous confidence intervals for all pairwise comparisons of the means of delta-lognormal distributions with application to rainfall data
Source: PLoS One. 2021 Jul 6;16(7):e0253935. doi: 10.1371/journal.pone.0253935 (PMC8260007; doi:10.1371/journal.pone.0253935)
Supplement: S1 Abbreviations — (PDF) [file pone.0253935.s001.pdf]

AIC	Akaike Infirmation Criteria
BCI-M	Bayesian Credible Interval-based Mixed Prior
BCI-U	Bayesian Credible Interval-based Uniform Prior
CI	Confidence Interval
CLT	Central Limit Theorem
CP	Coverge Probability
FGCI	Fiducial Generalized Confidence Intreval
FGPQ	Fiducial Generalized Pivotal Quantity
GPQ	Genarlized Pivatal Quantity
HPD	Highest Posterior Density
LEP	Lower Error Probability
MOVER	Method of Variance Estimates Recovery
PB	Parametric Boostrap
RAL	Relative Average Length
SCI	Simultaneous Confidence Interval
UEP	Upper Error Probability
