# Supplementary material for: Simultaneous confidence intervals for all pairwise comparisons of the means of delta-lognormal distributions with application to rainfall data
Source: PLoS One. 2021 Jul 6;16(7):e0253935. doi: 10.1371/journal.pone.0253935 (PMC8260007; doi:10.1371/journal.pone.0253935)
Supplement: S1 Appendix — (PDF) [file pone.0253935.s002.pdf]

Theorem 1. *Assume that* $W_j = (W_{j1}, W_{j,}, .., W_{jn_j}) \sim \Delta(\mu_j, \sigma_j^2, \delta_j)$; $j = 1, 2, ..., k$. *For* $w_j > 0, Y_j = \ln W_j \sim N(\mu_j, \sigma_j^2)$. *Let* θ_j *and* θ_l *be the means based on the* j^{th} *and* l^{th} *samples, respectively, where* $j, l = 1, 2, ..., k$ *and* $j \neq l$. *Let* θ_{jl} *be the difference between of* θ_j *and* θ_l. *Given an observation,* $\hat{\theta}_{jl}$ *be the estimate of* θ_{jl}; $\hat{\theta}_j$ *and* $\hat{\theta}_l$ *are the estimates of* θ_j *and* θ_l*, respectively. Also, let* $V_{\hat{\theta}_{jl}}$ *be the estimated variance of* $\hat{\theta}_{jl}$*, then*

$$P\left(L_{\theta_{jl}}^{(PB)} < \theta_{jl} < U_{\theta_{jl}}^{(PB)}\right) \to 1 - \alpha \tag{1}$$

where $\left[L_{\theta_{jl}}^{(PB)}, U_{\theta_{jl}}^{(PB)}\right] = \left[\hat{\theta}_{jl} - q_\alpha^{(PB)}\sqrt{V_{\hat{\theta}_{jl}}}, \hat{\theta}_{jl} + q_\alpha^{(PB)}\sqrt{V_{\hat{\theta}_{jl}}}\right]$.

Proof. Let $Var(\hat{\theta}_{jl})$ be the variance of $\hat{\theta}_{jl} = \hat{\theta}_j - \hat{\theta}_l$; $j, l = 1, 2, ..., k$ and $j \neq l$. Also, let $V_{\hat{\theta}_{jl}}$ be an estimate of $Var(\hat{\theta}_{jl})$. It is well known that

$$\begin{aligned} P\left(\hat{\theta}_{jl} - q_\alpha^{(PB)}\sqrt{V_{\hat{\theta}_{jl}}} < \theta_{jl} < \hat{\theta}_{jl} + q_\alpha^{(PB)}\sqrt{V_{\hat{\theta}_{jl}}}\right) &= P\left(\max_{j\neq l}\left|\frac{\hat{\theta}_{jl} - \theta_{jl}}{\sqrt{V_{\hat{\theta}_{jl}}}}\right| \leqslant q_\alpha^{(PB)}\right) \\ &= P\left(Q_n \leq q_\alpha^{(PB)}\right) \end{aligned} \tag{2}$$

Let $n = n_1 + n_2 + ... + n_k$ and $n_j/n \to \tau_j \in (0, 1)$ as $n \to \infty$ By the central limit theorem (CLT), we obtain that $n(\hat{\theta}_j - \theta_j)$ converges in distribution to Z , denoted as

$$n(\hat{\theta}_j - \theta_j) \xrightarrow{d} Z \tag{3}$$

where $Z = (Z_1, Z_2, ..., Z_k) \sim N(0, \sigma_j^2/\tau_j)$. Using Slutsky's theorem, Q_n converges in distribution to Q, denoted as $Q_n \xrightarrow{d} Q$ where

$$Q = \max_{j\neq l}\left|\frac{Z_j - Z_l}{\sqrt{\frac{\sigma_j^2}{\tau_j} + \frac{\sigma_l^2}{\tau_l}}}\right| \tag{4}$$

Using Skorokhod's theorem, let Y_n and Y be a random variable on a common probability space with a distribution Q_n and Q, receptively, so that $Y_n \xrightarrow{d} Y$ and $Q_n \xrightarrow{d} Q$. Assume that Z_j and Z_j^* are independent and identically distributed random variables such that

$$T(\boldsymbol{Y}, \boldsymbol{Y^*}, \boldsymbol{\mu}, \boldsymbol{\sigma^2}, \boldsymbol{\delta}) \to Q^* \tag{5}$$

where $Q^* = \max_{j\neq l}\left|\frac{Z_j^* - Z_l^*}{\sqrt{\frac{\sigma_j^2}{\tau_j} + \frac{\sigma_l^2}{\tau_l}}}\right|$. Suppose that $T(\boldsymbol{Y}, \boldsymbol{Y^*}, \boldsymbol{\mu}, \boldsymbol{\sigma^2}, \boldsymbol{\delta})$ be a continuous distribution and convergence in distribution to Q^*, and assume that $q_{\alpha(n)}^{(PB)} \to q_{1-\alpha}$; $q_{1-\alpha}$ stands for $(1-\alpha)^{th}$ percentile of Q^*. Then,

$$P\left(Q_n \leq q_{\alpha(n)}^{(PB)}\right) \to P\left(Q \leq q_{1-\alpha}\right) = P(Q^* \leq q_{1-\alpha}) = 1 - \alpha \tag{6}$$

as $n \to \infty$. Therefore, the $100(1-\alpha)\%$SCI-based PB for θ_{jl} has a asymptotically coverage probability, i.e, for all $j \neq l$

$$P\left(\theta_{jl} \in \left[\hat{\theta}_{jl} \mp q_\alpha^{(PB)}\sqrt{V_{\hat{\theta}_{jl}}}\right]\right) \to 1 - \alpha \tag{7}$$

It can be implied that
$P\left(L_{\theta_{jl}}^{(PB)} < \theta_{jl} < U_{\theta_{jl}}^{(PB)}\right) = P\left(\hat{\theta}_{jl} - q_{\alpha}^{(PB)}\sqrt{V_{\hat{\theta}_{jl}}} < \theta_{jl} < \hat{\theta}_{jl} + q_{\alpha}^{(PB)}\sqrt{V_{\hat{\theta}_{jl}}}\right) \to 1-\alpha;$
as $n \to \infty$.

□

Theorem 2. *Let $W_j = (W_{j1}, W_{j,}, .., W_{jn_j})$ be independent and identically distributed random variables with delta-lognormal distribution $\Delta(\mu_j, \sigma_j^2, \delta_j)$. For $w_j = 0$, $\delta_j = E(n_{j(0)}/n_j)$. For $w_j > 0$, $Y_j = \ln W_j$ where $\mu_j = E(y_j)$, $\sigma_j^2 = Var(y_j) > 0$. For $j, l = 1, 2, ..., k$ and $j \neq l$, let $\theta_{jl} = \theta_j - \theta_l$ where θ_j and θ_l be the means of W_j and W_l, respectively. Given an observation $w_j = (w_{j1}, w_{j,}, .., w_{jn_j})$, let $\hat{\theta}_j$ and $\hat{\theta}_l$ be the estimates of θ_j and θ_l, respectively. Also, let $V_{\hat{\theta}_{jl}}$ be the estimated variance of $\hat{\theta}_{jl} = \hat{\theta}_j - \hat{\theta}_l$. Let $n = \sum_{j=1}^{k} n_j$ where n_j be the sample size on j^{th} sample. Assume that $n_j/n \to \tau_j \in (0,1)$ as $n \to \infty$, then the $100(1-\alpha)\%$SFGCI has a asymptotically coverage probability, i.e, for all $j \neq l$*

$$P\left(L_{\theta_{jl}}^{(FGCI)} < \theta_{jl} < U_{\theta_{jl}}^{(FGCI)}\right) \to 1-\alpha \tag{8}$$

where $\left[L_{\theta_{jl}}^{(FGCI)}, U_{\theta_{jl}}^{(FGCI)}\right] = \left[\hat{\theta}_{jl} \mp t_{\alpha}^{(FGCI)}\sqrt{V_{\hat{\theta}_{jl}}}\right]$.

Proof. This is also followed from Theorem 1. Define $\theta_j = (\mu_j, \sigma_j^2, \delta_j)$ and $\theta_l = (\mu_l, \sigma_l^2, \delta_l)$ so that $Var(\hat{\theta}_{jl})$ be the variance of the difference betweeen $\hat{\theta}_j$ and $\hat{\theta}_l$. Also, $V_{\hat{\theta}_{jl}}$ be an estimate of $Var(\hat{\theta}_{jl})$. For all $j \neq l$,

$$\begin{aligned} P\left(L_{\theta_{jl}}^{(FGCI)} < \theta_{jl} < U_{\theta_{jl}}^{(FGCI)}\right) &= P\left(\theta_{ij} \in \left[\hat{\theta}_{jl} \mp t_{1-\alpha}^{(FGCI)}\sqrt{V_{\hat{\theta}_{jl}}}\right]\right) \\ &= P\left(\underset{j\neq l}{max}\left|\frac{\hat{\theta}_{jl}-\theta_{jl}}{\sqrt{V_{\hat{\theta}_{jl}}}}\right| \leqslant t_{\alpha}\right) \\ &= P\left(Q_n \leq t_{\alpha}\right) \end{aligned} \tag{9}$$

as $n \to \infty$. It can be implied that
$P\left(\hat{\theta}_{jl} - t_{\alpha}^{(FGCI)}\sqrt{V_{\theta_{jl}}} < \theta_{jl} < \hat{\theta}_{jl} + t_{\alpha}^{(FGCI)}\sqrt{V_{\theta_{jl}}}\right) \to 1-\alpha$ for all $j \neq l$. □

Theorem 3. *Let $W_j = (W_{j1}, W_{j,}, .., W_{jn_j}) \sim \Delta(\mu_j, \sigma_j^2, \delta_j)$ so that $Y_j = \ln W_j \sim N(\mu_j, \sigma_j^2)$; $w_j > 0$. Also, let (θ_j, θ_l) and (β_j, β_l) be the delta-lognormal means and log-transformed delta-lognormal means based on j^{th} and l^{th} samples, respectively. Given an observation, let $\hat{\theta}_{jl} = \hat{\theta}_j - \hat{\theta}_l = \exp(\hat{\beta}_j) - \exp(\hat{\beta}_l)$ where $(\hat{\theta}_j, \hat{\theta}_l)$ and $(\hat{\beta}_j, \hat{\beta}_l)$ be the estimates of (θ_j, θ_l) and (β_j, β_l), respectively. The $SCI_{\theta_{ji}}^{(MOVER)} = \left[L_{\theta_{jl}}^{(MOVER)}, U_{\theta_{jl}}^{(MOVER)}\right]$ becomes the $100(1-\alpha)\%$SCIs for θ_{jl} based on MOVER where*

$$\begin{aligned} L_{\theta_{jl}}^{(MOVER)} &= \hat{\theta}_{jl} - \sqrt{(\hat{\theta}_j - l_{\theta_j})^2 + (u_{\theta_l} - \hat{\theta}_l)^2} \\ U_{\theta_{jl}}^{(MOVER)} &= \hat{\theta}_{jl} + \sqrt{(u_{\theta_j} - \hat{\theta}_j)^2 + (\hat{\theta}_l - l_{\theta_l})^2} \end{aligned} \tag{10}$$

where $\left[l_{\theta_j}, u_{\theta_j}\right] = \left[\exp\left\{l_{\beta_j}\right\}, \exp\left\{u_{\beta_j}\right\}\right]$ and $= [l_{\theta_l}, u_{\theta_l}] = [\exp\{l_{\beta_l}\}, \exp\{u_{\beta_l}\}]$. Then, $P\left(L_{\theta_{jl}}^{(MOVER)} < \theta_{jl} < U_{\theta_{jl}}^{(MOVER)}\right) \to 1-\alpha$; $j, l = 1, 2, ..., k$ and $j \neq l$.

Proof. Assume that $(l_{\beta_j}, u_{\beta_j})$ and $(l_{\beta_l}, u_{\beta_l})$ be the $100(1-\alpha)\%$CIs for β_j and β_l, respectively. By CLT, the estimates $\hat{\beta}_j$ and $\hat{\beta}_l$ are independent such that the upper limit for $\beta_{j+l} = \beta_j + \beta_l$, given by

$$U_{\beta_{j+l}} = (\hat{\beta}_j + \hat{\beta}_l) + v_{\alpha/2}\sqrt{v\hat{a}r(\hat{\beta}_j) + v\hat{a}r(\hat{\beta}_l)} \tag{11}$$

where v_α stands for the αth percentile of $N(0,1)$. Notice that $U_{\beta_{j+l}}$ be closer to $u_{\beta_j} + u_{\beta_l}$ than $\hat{\beta}_j + \hat{\beta}_l$ so that the estimate variance at $\beta_j = u_{\beta_j}$ can be recovered from u_{β_j} using CLT as $v\hat{a}r(\hat{\beta}_j) = (u_{\beta_j} - \hat{\beta}_j)^2/v^2_{\alpha/2}$ and $v\hat{a}r(\hat{\beta}_l) = (u_{\beta_l} - \hat{\beta}_l)^2/v^2_{\alpha/2}$. From Eq (11), we obtain that

$$U_{\beta_{j+l}} = (\hat{\beta}_j + \hat{\beta}_l) + \sqrt{(u_{\beta_j} - \hat{\beta}_j)^2 + (u_{\beta_l} - \hat{\beta}_l)^2} \tag{12}$$

Similarly, $L_{\beta_{j+l}} = (\hat{\beta}_j + \hat{\beta}_l) - \sqrt{(\hat{\beta}_j - l_{\beta_j})^2 + (\hat{\beta}_l - l_{\beta_l})^2}$. For focusing on SCIs for $\theta_{jl} = \theta_j - \theta_l$, the $\left[-u_{\theta_j}, -l_{\theta_j}\right]$ becomes the CIs for $-\theta_j$ where the estimate variance of θ_j are $v\hat{a}r(\hat{\theta}_j) = (u_{\theta_j} - \hat{\theta}_j)^2/v^2_{\alpha/2}$ and $v\hat{a}r(\hat{\theta}_j) = (\hat{\theta}_j - l_{\theta_j})^2/v^2_{\alpha/2}$ for lower and upper limits for $-\theta_j$, respectively. Then, SCIs for θ_{jl} is defined as

$$\begin{aligned} SCI^{(MOVER)}_{\theta_{jl}} &= \left[L^{(MOVER)}_{\theta_{ij}}, U^{(MOVER)}_{\theta_{jl}}\right] \\ &= \left[\hat{\theta}_{jl} - \sqrt{(\hat{\theta}_j - l_{\theta_j})^2 + (u_{\theta_l} - \hat{\theta}_l)^2}, \hat{\theta}_{jl} + \sqrt{(u_{\theta_j} - \hat{\theta}_j)^2 + (\hat{\theta}_l - l_{\theta_l})^2}\right] \end{aligned} \tag{13}$$

For examining the coverage probability, we obtain that

$$\begin{aligned} P\left(L^{(MOVER)}_{\theta_{jl}} < \theta_{jl} < U^{(MOVER)}_{\theta_{jl}}\right) &= P\left(\theta_{jl} \in \left\{\hat{\theta}_{jl} \pm v_{\alpha/2}\sqrt{var(\hat{\theta}_j) + var(\hat{\theta}_l)}\right\}; \forall j \neq l\right) \\ &= P\left(\max_{j\neq l}\left|\frac{\hat{\theta}_{jl} - \theta_{jl}}{\sqrt{v\hat{a}r(\hat{\theta}_j - \hat{\theta}_l)}}\right| \leqslant v_{\alpha/2}\right) \\ &= P(T_n \leqslant v_{\alpha/2}) \end{aligned} \tag{14}$$

Let $n = n_1 + n_2 + ... + n_k$ and $\tau_j = \lim_{n\to\infty} n_j/n$ exits; $\tau_j \in (0,1)$. By CLT, we obtain

$$v_j = n(\hat{\theta}_j - \theta_j) \xrightarrow{d} N(0, \sigma_j^2/\tau_j) \tag{15}$$

Then, $T = \max_{j\neq l}\left|\frac{v_j - v_l}{\sqrt{\frac{\sigma_j^2}{\tau_j} + \frac{\sigma_l^2}{\tau_l}}}\right|$. Using Slutsky's theorem, $Y_n \to Y$ and $T_n \to T$ as $n \to \infty$.

Let $T^* = \max_{j\neq l}\left|\frac{v_j^* - v_l^*}{\sqrt{\frac{\sigma_j^2}{\tau_j} + \frac{\sigma_l^2}{\tau_l}}}\right|$. Apply Skorokhod's theorem, obtain that

$$T(\boldsymbol{Y}, \boldsymbol{Y^*}, \boldsymbol{\mu}, \boldsymbol{\sigma^2}, \boldsymbol{\delta}) \to T^* \tag{16}$$

According to the definition of convergence in distribution, this can be implied that $v_{\alpha/2}(Y) \to t_{\alpha/2}$; $t_{\alpha/2}$ stands for the $(\alpha/2)^{th}$ percentile of the distribution of T^*. Then,

$$P(T_n \leqslant v_{\alpha/2}) \to P(T \leqslant t_{\alpha/2}) \tag{17}$$

which is equivalent to $P(T^* \leqslant t_{\alpha/2}) = 1 - \alpha$; as $n \to \infty$. This can be implied that $P\left(L_{\theta_{jl}}^{(MOVER)} < \theta_{jl} < U_{\theta_{jl}}^{(MOVER)}\right) \to 1 - \alpha$; $j \neq l$. □
