# Supplementary figures and images for: Simultaneous confidence intervals for all pairwise comparisons of the means of delta-lognormal distributions with application to rainfall data
Source: PLoS One. 2021 Jul 6;16(7):e0253935. doi: 10.1371/journal.pone.0253935 (PMC8260007; doi:10.1371/journal.pone.0253935)

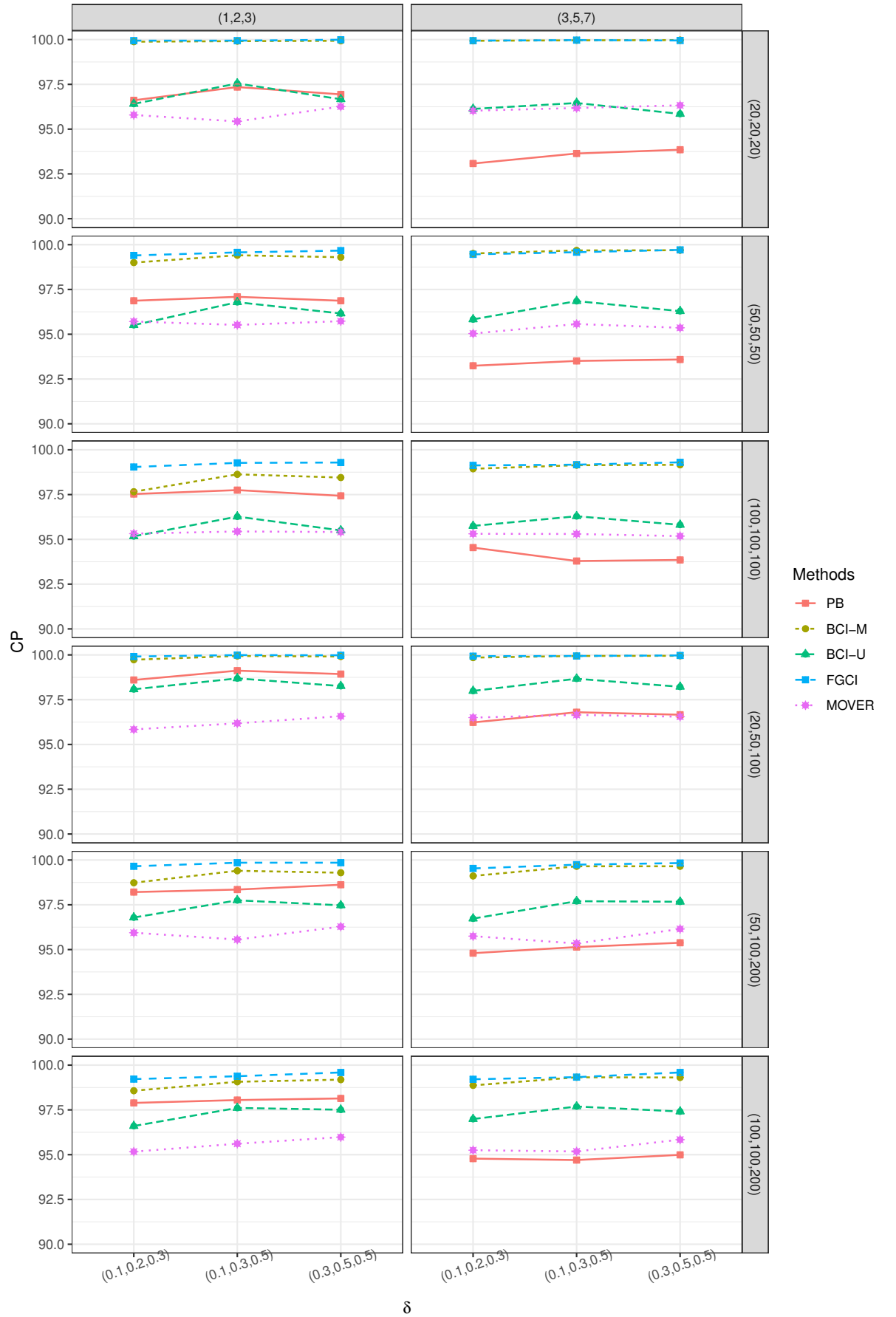

Supplement: S1 Fig — (PDF) [file pone.0253935.s005.pdf]

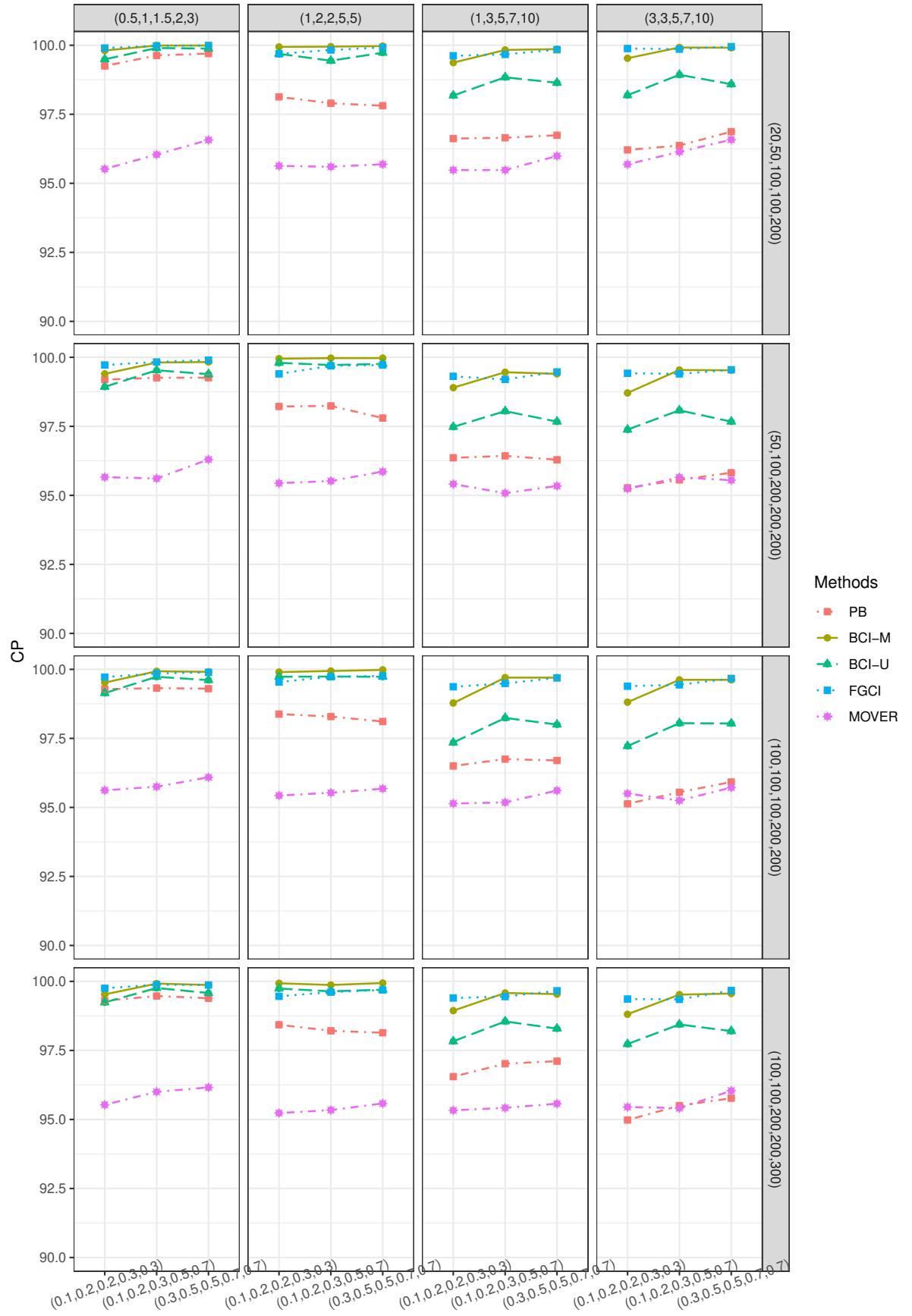

$\delta$

Supplement: S2 Fig — (PDF) [file pone.0253935.s006.pdf]

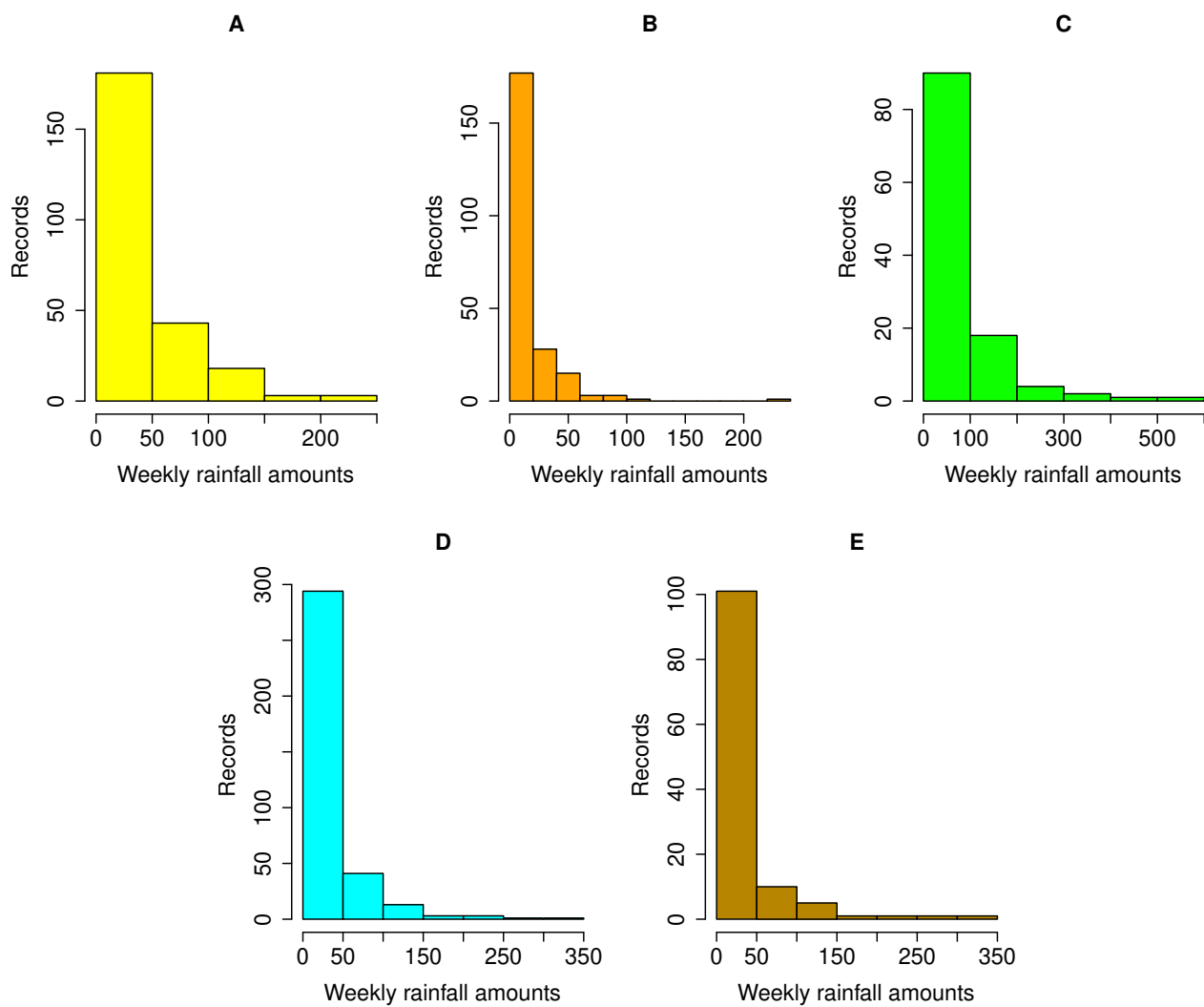

Supplement: S3 Fig — (A) Northern, (B) Central, (C) Eastern, (D) Southeastern, and (E) Southwestern. (PDF) [file pone.0253935.s007.pdf]

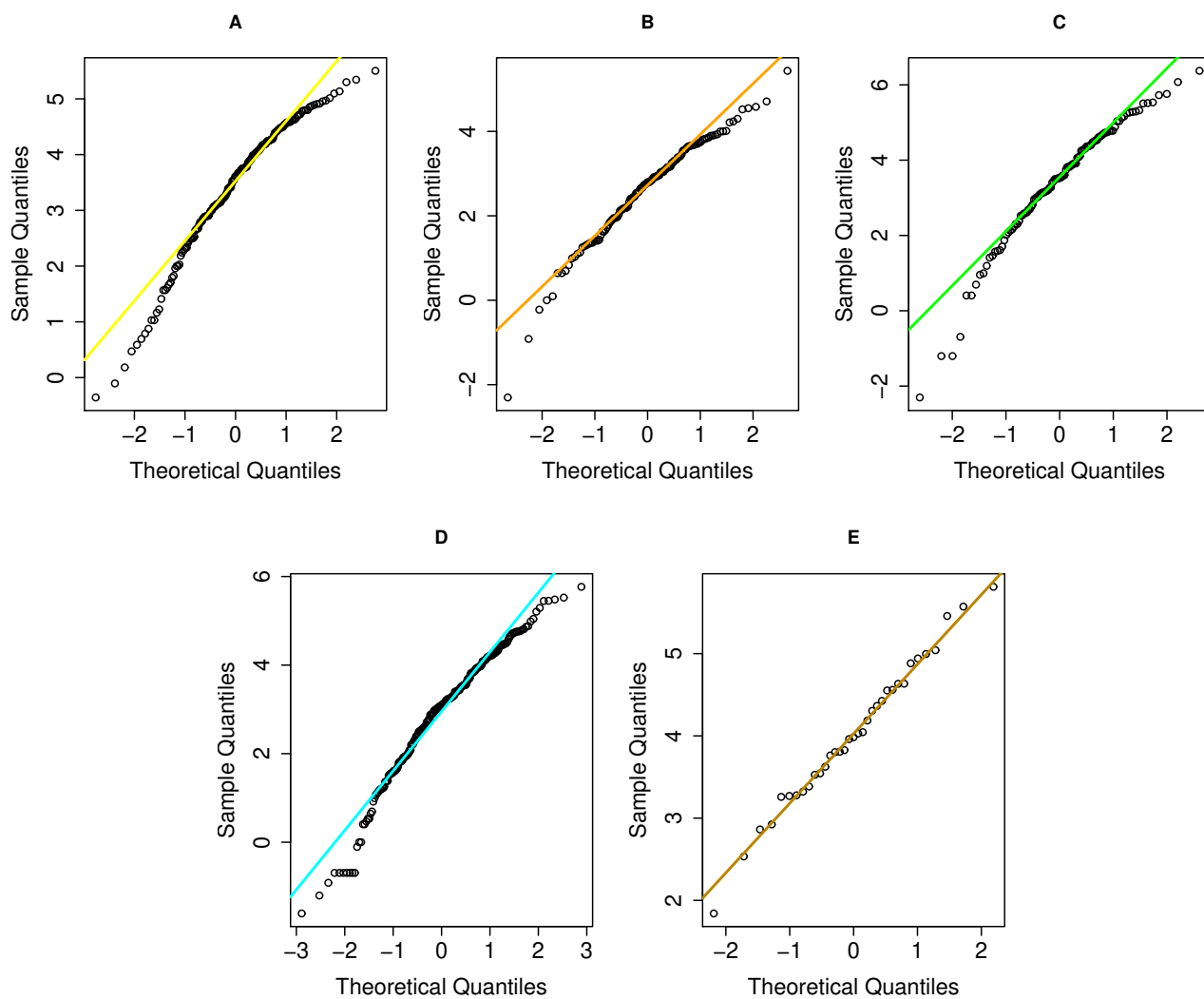

Supplement: S4 Fig — (A) Northern (B) Central (C) Eastern (D) Southeastern (E) Southwestern. (PDF) [file pone.0253935.s008.pdf]
