# Supplementary material for: Simultaneous confidence intervals for all pairwise comparisons of the means of delta-lognormal distributions with application to rainfall data
Source: PLoS One. 2021 Jul 6;16(7):e0253935. doi: 10.1371/journal.pone.0253935 (PMC8260007; doi:10.1371/journal.pone.0253935)
Supplement: S1 Table — (PDF) [file pone.0253935.s009.pdf]

Sample case $k = 3$			PB			BCI-M			BCI-U			FGCI			MOVER			RAL				
**n**	σ^2	δ	LEP	CP	UEP	LEP	CP	UEP	LEP	CP	UEP	LEP	CP	UEP	LEP	CP	UEP	PB	BCI-M	BCI-U	FGCI	MOVER
(20,20,20)	(1,2,3)	(0.1,0.2,0.3)	3.39	96.61	0.00	0.12	99.88	0.00	3.59	96.41	0.00	0.06	99.94	0.00	2.02	95.79	2.19	0.214	1.351	**0.201**	1.204	*
		(0.1,0.3,0.5)	2.65	97.35	0.00	0.09	99.91	0.00	2.45	97.55	0.00	0.06	99.94	0.00	1.94	95.43	2.63	0.004	0.308	**0.004**	0.221	*
		(0.3,0.5,0.5)	3.06	96.94	0.00	0.07	99.93	0.00	3.33	96.67	0.00	0.01	99.99	0.00	1.80	96.26	1.94	2.7e-4	0.080	**2.8e-4**	0.052	*
	(3,5,7)	(0.1,0.2,0.3)	6.92	93.08	0.00	0.07	99.93	0.00	3.87	96.13	0.00	0.06	99.94	0.00	2.42	96.03	1.55	9.7e-5	0.182	**2.5e-4**	0.110	*
		(0.1,0.3,0.5)	6.36	93.64	0.00	0.04	99.96	0.00	3.54	96.46	0.00	0.03	99.97	0.00	2.16	96.18	1.66	1.6e-11	5e-4	**8.1e-11**	4e-4	*
		(0.3,0.5,0.5)	6.15	93.85	0.00	0.03	99.97	0.00	4.15	95.85	0.00	0.05	99.95	0.00	2.14	96.33	1.53	8.4e-12	0.001	**6.0e-11**	0.001	*
(50,50,50)	(1,2,3)	(0.1,0.2,0.3)	3.13	96.87	0.00	1.00	99.00	0.00	4.49	95.51	0.00	0.60	99.40	0.00	1.96	95.71	2.33	1.031	1.709	**0.952**	1.762	*
		(0.1,0.3,0.5)	2.91	97.09	0.00	0.59	99.41	0.00	3.21	96.79	0.00	0.43	99.57	0.00	1.95	95.52	2.53	0.828	1.773	**0.805**	1.740	*
		(0.3,0.5,0.5)	3.13	96.87	0.00	0.70	99.30	0.00	3.84	96.16	0.00	0.33	99.67	0.00	1.78	95.73	2.49	0.810	1.786	**0.788**	1.805	*
	(3,5,7)	(0.1,0.2,0.3)	6.76	93.24	0.00	0.49	99.51	0.00	4.17	95.83	0.00	0.54	99.46	0.00	2.49	95.04	2.47	0.338	1.793	**0.544**	1.571	*
		(0.1,0.3,0.5)	6.49	93.51	0.00	0.32	99.68	0.00	3.15	96.85	0.00	0.42	99.58	0.00	2.05	95.57	2.38	0.096	1.257	**0.178**	0.979	*
		(0.3,0.5,0.5)	6.41	93.59	0.00	0.31	99.69	0.00	3.71	96.29	0.00	0.29	99.71	0.00	2.19	95.36	2.45	0.088	1.226	**0.169**	1.056	*
(100,100,100)	(1,2,3)	(0.1,0.2,0.3)	2.47	97.53	0.00	2.34	97.66	0.00	4.81	95.17	0.02	0.96	99.04	0.00	1.96	95.32	2.72	1.242	1.522	1.110	1.632	**
		(0.1,0.3,0.5)	2.25	97.75	0.00	1.37	98.63	0.00	3.71	96.27	0.03	0.73	99.27	0.00	1.75	95.44	2.81	1.152	1.590	1.061	1.662	**
		(0.3,0.5,0.5)	2.57	97.43	0.00	1.55	98.45	0.00	4.49	95.50	0.01	0.71	99.29	0.00	1.79	95.41	2.80	1.147	1.617	1.071	1.702	**
	(3,5,7)	(0.1,0.2,0.3)	5.46	94.54	0.00	1.06	98.94	0.00	4.25	95.75	0.00	0.87	99.13	0.00	2.24	95.31	2.45	0.862	2.068	**1.229**	1.907	*
		(0.1,0.3,0.5)	6.21	93.79	0.00	0.86	99.14	0.00	3.71	96.29	0.00	0.83	99.17	0.00	2.25	95.30	2.45	0.629	2.048	**0.975**	1.767	*
		(0.3,0.5,0.5)	6.15	93.85	0.00	0.84	99.16	0.00	4.19	95.81	0.00	0.70	99.30	0.00	2.05	95.18	2.77	0.611	2.040	**0.954**	1.860	*
(20,50,100)	(1,2,3)	(0.1,0.2,0.3)	1.40	98.60	0.00	0.27	99.73	0.00	1.91	98.08	0.01	0.09	99.91	0.00	2.07	95.84	2.09	0.887	1.202	**0.824**	1.496	**
		(0.1,0.3,0.5)	0.88	99.12	0.00	0.06	99.94	0.00	1.31	98.69	0.00	0.01	99.99	0.00	1.58	96.18	2.24	0.824	1.252	**0.793**	1.526	**
		(0.3,0.5,0.5)	1.07	98.93	0.00	0.09	99.91	0.00	1.74	98.26	0.00	0.02	99.98	0.00	1.73	96.58	1.69	0.767	1.262	**0.733**	1.557	**
	(3,5,7)	(0.1,0.2,0.3)	3.77	96.23	0.00	0.15	99.85	0.00	2.01	97.99	0.00	0.07	99.93	0.00	2.07	96.50	1.43	**0.500**	1.501	0.726	1.785	*
		(0.1,0.3,0.5)	3.20	96.80	0.00	0.06	99.94	0.00	1.33	98.67	0.00	0.06	99.94	0.00	2.05	96.65	1.30	**0.413**	1.590	0.637	1.848	*
		(0.3,0.5,0.5)	3.34	96.66	0.00	0.05	99.95	0.00	1.78	98.22	0.00	0.03	99.97	0.00	2.21	96.55	1.24	**0.217**	1.165	0.329	1.387	*
(50,100,200)	(1,2,3)	(0.1,0.2,0.3)	1.79	98.21	0.00	1.27	98.73	0.01	2.99	96.79	0.22	0.35	99.65	0.00	1.80	95.94	2.26	1.101	1.163	**0.977**	1.439	**
		(0.1,0.3,0.5)	1.65	98.35	0.00	0.60	99.40	0.00	2.17	97.75	0.09	0.15	99.85	0.00	1.73	95.56	2.71	1.047	1.181	**0.948**	1.449	**
		(0.3,0.5,0.5)	1.38	98.62	0.00	0.71	99.29	0.00	2.50	97.47	0.03	0.15	99.85	0.00	1.60	96.28	2.12	1.032	1.202	**0.941**	1.475	**
	(3,5,7)	(0.1,0.2,0.3)	5.20	94.80	0.00	0.89	99.11	0.00	3.27	96.73	0.00	0.47	99.53	0.00	2.08	95.75	2.17	**0.909**	1.687	1.249	1.839	*
		(0.1,0.3,0.5)	4.86	95.14	0.00	0.35	99.65	0.00	2.30	97.70	0.00	0.26	99.74	0.00	2.28	95.34	2.38	**0.816**	1.740	1.176	1.847	*
		(0.3,0.5,0.5)	4.62	95.38	0.00	0.35	99.65	0.00	2.33	97.67	0.00	0.17	99.83	0.00	1.93	96.15	1.92	**0.776**	1.715	1.111	1.921	*
(100,100,200)	(1,2,3)	(0.1,0.2,0.3)	2.11	97.89	0.00	1.43	98.57	0.00	3.25	96.60	0.15	0.78	99.22	0.00	2.05	95.17	2.78	1.229	1.248	1.040	1.519	**
		(0.1,0.3,0.5)	1.95	98.05	0.00	0.93	99.07	0.00	2.34	97.61	0.05	0.62	99.38	0.00	1.75	95.61	2.64	1.177	1.275	1.011	1.543	**
		(0.3,0.5,0.5)	1.86	98.14	0.00	0.81	99.19	0.00	2.47	97.51	0.02	0.41	99.59	0.00	1.65	95.98	2.37	1.165	1.306	1.014	1.578	**
	(3,5,7)	(0.1,0.2,0.3)	5.22	94.78	0.00	1.13	98.87	0.00	3.01	96.99	0.00	0.79	99.21	0.00	2.10	95.25	2.65	**1.041**	1.816	1.350	1.949	*
		(0.1,0.3,0.5)	5.30	94.70	0.00	0.68	99.32	0.00	2.31	97.69	0.00	0.67	99.33	0.00	2.26	95.18	2.56	**0.934**	1.855	1.255	1.947	*
		(0.3,0.5,0.5)	5.01	94.99	0.00	0.69	99.31	0.00	2.59	97.41	0.00	0.41	99.59	0.00	1.85	95.84	2.31	**0.889**	1.866	1.213	2.040	*

Note: Bold denotes the best-performing method. *MOVER satisfies the CP criteria, and **it is the best-performing method.
