# Supplementary material for: Simultaneous confidence intervals for all pairwise comparisons of the means of delta-lognormal distributions with application to rainfall data
Source: PLoS One. 2021 Jul 6;16(7):e0253935. doi: 10.1371/journal.pone.0253935 (PMC8260007; doi:10.1371/journal.pone.0253935)
Supplement: S2 Table — (PDF) [file pone.0253935.s010.pdf]

Table 1. CPs, LEPs, UEPs and RALs of 95% SCIs for θ_{jl}: 5 sample cases.

Sample case $k=5$			PB			BCI-M			BCI-U			FGCI			MOVER			RAL				
**n**	$\boldsymbol{\sigma^2}$	$\boldsymbol{\delta}$	LEP	CP	UEP	LEP	CP	UEP	LEP	CP	UEP	LEP	CP	UEP	LEP	CP	UEP	PB	BCI-M	BCI-U	FGCI	MOVER
(20,50,100,100,200)	(0.5,1,1.5,2,3)	(0.1,0.2,0.2,0.3,0.3)	0.75	99.25	0.00	0.19	99.81	0.00	0.50	99.49	0.01	0.10	99.90	0.00	2.14	95.52	2.34	1.170	1.628	1.373	1.586	**
		(0.1,0.2,0.3,0.5,0.7)	0.36	99.63	0.01	0.01	99.99	0.00	0.10	99.90	0.00	0.02	99.98	0.00	1.67	96.04	2.28	1.052	1.669	1.246	1.616	**
		(0.3,0.5,0.5,0.7,0.7)	0.30	99.70	0.00	0.01	99.99	0.00	0.12	99.88	0.00	0.01	99.99	0.00	1.54	96.57	1.89	0.993	1.733	1.197	1.698	**
	(1,2,2,5,5)	(0.1,0.2,0.2,0.3,0.3)	1.87	98.13	0.00	0.06	99.94	0.00	0.31	99.69	0.00	0.31	99.69	0.00	2.11	95.63	2.26	1.064	2.242	1.543	1.801	**
		(0.1,0.2,0.3,0.5,0.7)	2.09	97.90	0.01	0.05	99.95	0.00	0.55	99.44	0.01	0.17	99.83	0.00	2.06	95.60	2.35	**0.876**	2.357	1.346	1.808	**
		(0.3,0.5,0.5,0.7,0.7)	2.19	97.81	0.00	0.03	99.97	0.00	0.27	99.73	0.00	0.08	99.92	0.00	2.16	95.69	2.15	**0.581**	2.330	0.940	1.649	*
	(1,3,5,7,10)	(0.1,0.2,0.2,0.3,0.3)	3.38	96.62	0.00	0.63	99.37	0.00	1.82	98.18	0.00	0.38	99.62	0.00	2.22	95.48	2.30	**0.956**	2.829	1.981	2.143	*
		(0.1,0.2,0.3,0.5,0.7)	3.35	96.65	0.00	0.17	99.83	0.00	1.16	98.84	0.00	0.33	99.67	0.00	2.15	95.48	2.37	**0.451**	2.528	1.097	1.584	*
		(0.3,0.5,0.5,0.7,0.7)	3.26	96.74	0.00	0.14	99.86	0.00	1.36	98.64	0.00	0.16	99.84	0.00	1.90	95.99	2.11	**0.349**	2.303	0.833	1.789	*
	(3,3,5,7,10)	(0.1,0.2,0.2,0.3,0.3)	3.79	96.21	0.00	0.47	99.53	0.00	1.81	98.19	0.00	0.12	99.88	0.00	2.30	95.69	2.01	**0.798**	2.723	1.851	2.572	*
		(0.1,0.2,0.3,0.5,0.7)	3.63	96.37	0.00	0.08	99.92	0.00	1.07	98.93	0.00	0.14	99.86	0.00	2.05	96.14	1.82	**0.387**	2.517	1.065	1.884	*
		(0.3,0.5,0.5,0.7,0.7)	3.13	96.87	0.00	0.09	99.91	0.00	1.41	98.59	0.00	0.05	99.95	0.00	1.85	96.58	1.58	**0.312**	2.335	0.796	2.260	*
(50,100,200,200,200)	(0.5,1,1.5,2,3)	(0.1,0.2,0.2,0.3,0.3)	0.79	99.19	0.02	0.60	99.40	0.00	1.06	98.93	0.01	0.28	99.72	0.00	1.92	95.66	2.42	1.257	1.812	1.570	1.495	**
		(0.1,0.2,0.3,0.5,0.7)	0.74	99.26	0.00	0.19	99.81	0.00	0.47	99.53	0.00	0.17	99.83	0.00	1.78	95.61	2.61	1.145	1.862	1.413	1.531	**
		(0.3,0.5,0.5,0.7,0.7)	0.74	99.26	0.00	0.17	99.83	0.00	0.61	99.39	0.00	0.10	99.90	0.00	1.61	96.30	2.09	1.115	1.914	1.420	1.564	**
	(1,2,2,5,5)	(0.1,0.2,0.2,0.5,0.6)	1.78	98.22	0.01	0.05	99.95	0.00	0.20	99.80	0.00	0.60	99.40	0.00	2.16	95.44	2.40	1.204	2.132	1.703	1.662	**
		(0.1,0.2,0.5,0.5,0.6)	1.76	98.24	0.01	0.03	99.97	0.00	0.27	99.72	0.00	0.31	99.69	0.00	1.85	95.52	2.63	1.043	2.274	1.531	1.708	**
		(0.1,0.5,0.5,0.5,0.6)	2.19	97.80	0.00	0.03	99.97	0.00	0.24	99.76	0.00	0.28	99.72	0.00	1.91	95.86	2.23	**0.960**	2.342	1.437	1.750	*
	(1,3,5,7,10)	(0.1,0.2,0.2,0.5,0.6)	3.64	96.36	0.00	1.10	98.90	0.00	2.52	97.48	0.00	0.69	99.31	0.00	2.13	95.41	2.46	**0.926**	3.098	2.247	1.771	*
		(0.1,0.2,0.5,0.5,0.6)	3.57	96.43	0.00	0.54	99.46	0.00	1.95	98.05	0.00	0.80	99.20	0.00	2.31	95.08	2.62	**0.403**	2.603	1.141	1.261	*
		(0.1,0.5,0.5,0.5,0.6)	3.71	96.29	0.00	0.60	99.40	0.00	2.33	97.67	0.00	0.53	99.47	0.00	2.18	95.34	2.47	**0.423**	2.593	1.139	1.423	*
	(3,3,5,7,10)	(0.1,0.2,0.2,0.3,0.3)	4.73	95.27	0.00	1.29	98.71	0.00	2.62	97.38	0.00	0.58	99.42	0.00	2.25	95.24	2.51	**0.816**	3.070	2.231	1.821	*
		(0.1,0.2,0.3,0.5,0.7)	4.44	95.56	0.00	0.46	99.54	0.00	1.92	98.08	0.00	0.60	99.40	0.00	2.07	95.65	2.28	**0.341**	2.578	1.112	1.265	*
		(0.3,0.5,0.5,0.7,0.7)	4.18	95.82	0.00	0.47	99.53	0.00	2.33	97.67	0.00	0.45	99.55	0.00	2.08	95.55	2.36	**0.365**	2.595	1.135	1.447	*
(100,100,100,200,200)	(0.5,1,1.5,2,3)	(0.1,0.2,0.2,0.5,0.6)	0.72	99.28	0.00	0.48	99.52	0.00	0.85	99.14	0.00	0.28	99.72	0.00	1.89	95.62	2.49	1.301	1.772	1.534	1.538	**
		(0.1,0.2,0.5,0.5,0.6)	0.68	99.32	0.00	0.07	99.93	0.00	0.27	99.73	0.00	0.15	99.85	0.00	1.70	95.75	2.55	1.200	1.822	1.390	1.581	**
		(0.1,0.5,0.5,0.5,0.6)	0.70	99.30	0.00	0.09	99.91	0.00	0.39	99.61	0.00	0.11	99.89	0.00	1.62	96.09	2.29	1.161	1.873	1.392	1.623	**
	(1,2,2,5,5)	(0.1,0.2,0.2,0.5,0.6)	1.62	98.38	0.00	0.10	99.90	0.00	0.27	99.73	0.00	0.46	99.54	0.00	2.03	95.43	2.54	1.245	2.111	1.690	1.699	**
		(0.1,0.2,0.5,0.5,0.6)	1.71	98.29	0.00	0.06	99.94	0.00	0.26	99.74	0.00	0.27	99.73	0.00	1.88	95.53	2.59	1.086	2.243	1.519	1.745	**
		(0.1,0.5,0.5,0.5,0.6)	1.89	98.11	0.00	0.02	99.98	0.00	0.27	99.73	0.00	0.24	99.76	0.00	1.93	95.68	2.40	**0.991**	2.325	1.427	1.786	*
	(1,3,5,7,10)	(0.1,0.2,0.2,0.5,0.6)	3.50	96.50	0.00	1.22	98.78	0.00	2.65	97.35	0.00	0.63	99.37	0.00	2.34	95.14	2.52	**0.989**	3.040	2.210	1.884	*
		(0.1,0.2,0.5,0.5,0.6)	3.25	96.75	0.00	0.30	99.70	0.00	1.76	98.24	0.00	0.51	99.49	0.00	2.19	95.18	2.64	**0.451**	2.602	1.151	1.333	*
		(0.1,0.5,0.5,0.5,0.6)	3.30	96.70	0.00	0.30	99.70	0.00	2.00	98.00	0.00	0.31	99.69	0.00	1.91	95.61	2.48	**0.472**	2.598	1.151	1.531	*
	(3,3,5,7,10)	(0.1,0.2,0.2,0.3,0.3)	4.87	95.13	0.00	1.19	98.81	0.00	2.78	97.22	0.00	0.61	99.39	0.00	2.20	95.50	2.30	**0.855**	3.033	2.204	1.856	*
		(0.1,0.2,0.3,0.5,0.7)	4.45	95.55	0.00	0.38	99.62	0.00	1.95	98.05	0.00	0.56	99.44	0.00	2.20	95.25	2.55	**0.358**	2.570	1.118	1.287	*
		(0.3,0.5,0.5,0.7,0.7)	4.08	95.92	0.00	0.38	99.62	0.00	1.96	98.04	0.00	0.33	99.67	0.00	1.86	95.72	2.42	**0.377**	2.576	1.123	1.479	*
(100,100,200,200,300)	(0.5,1,1.5,2,3)	(0.1,0.2,0.2,0.5,0.6)	0.70	99.30	0.00	0.47	99.53	0.00	0.76	99.24	0.00	0.25	99.75	0.00	1.91	95.53	2.56	1.300	1.685	1.530	1.495	**
		(0.1,0.2,0.5,0.5,0.6)	0.53	99.47	0.00	0.08	99.92	0.00	0.23	99.76	0.01	0.12	99.88	0.00	1.58	96.00	2.43	1.233	1.685	1.400	1.534	**
		(0.1,0.5,0.5,0.5,0.6)	0.61	99.39	0.00	0.13	99.87	0.00	0.41	99.58	0.00	0.13	99.87	0.00	1.66	96.16	2.18	1.194	1.739	1.408	1.573	**
	(1,2,2,5,5)	(0.1,0.2,0.2,0.5,0.6)	1.56	98.43	0.01	0.07	99.93	0.00	0.26	99.74	0.00	0.54	99.46	0.00	2.14	95.23	2.63	1.245	2.104	1.728	1.643	**
		(0.1,0.2,0.5,0.5,0.6)	1.78	98.21	0.01	0.13	99.87	0.00	0.35	99.64	0.01	0.39	99.61	0.00	2.04	95.34	2.62	1.152	2.234	1.638	1.701	**
		(0.1,0.5,0.5,0.5,0.6)	1.86	98.14	0.00	0.06	99.94	0.00	0.30	99.70	0.00	0.31	99.69	0.00	1.86	95.58	2.56	1.035	2.309	1.507	1.723	**
	(1,3,5,7,10)	(0.1,0.2,0.2,0.5,0.6)	3.45	96.55	0.00	1.06	98.94	0.00	2.17	97.83	0.00	0.60	99.40	0.00	2.20	95.33	2.48	1.105	3.064	2.467	1.918	**
		(0.1,0.2,0.5,0.5,0.6)	2.98	97.02	0.00	0.42	99.58	0.00	1.45	98.55	0.00	0.55	99.45	0.00	2.05	95.42	2.53	**0.727**	2.859	1.725	1.620	*
		(0.1,0.5,0.5,0.5,0.6)	2.89	97.11	0.00	0.46	99.54	0.00	1.71	98.29	0.00	0.34	99.66	0.00	1.87	95.57	2.56	**0.742**	2.840	1.680	1.840	*
	(3,3,5,7,10)	(0.1,0.2,0.2,0.3,0.3)	5.02	94.98	0.00	1.19	98.81	0.00	2.27	97.73	0.00	0.64	99.36	0.00	2.25	95.45	2.29	**0.944**	3.060	2.464	1.876	*
		(0.1,0.2,0.3,0.5,0.7)	4.50	95.50	0.00	0.48	99.52	0.00	1.56	98.44	0.00	0.65	99.35	0.00	2.19	95.41	2.40	**0.626**	2.843	1.725	1.613	*
		(0.3,0.5,0.5,0.7,0.7)	4.23	95.77	0.00	0.44	99.56	0.00	1.80	98.20	0.00	0.33	99.67	0.00	1.91	96.04	2.06	**0.614**	2.825	1.667	1.803	*

Note: Bold denotes the best-performing method. *MOVER satisfies the CP criteria, and **it also is the best-performing method.
