# Supplementary material for: Simultaneous confidence intervals for all pairwise comparisons of the means of delta-lognormal distributions with application to rainfall data
Source: PLoS One. 2021 Jul 6;16(7):e0253935. doi: 10.1371/journal.pone.0253935 (PMC8260007; doi:10.1371/journal.pone.0253935)
Supplement: S3 Table — (PDF) [file pone.0253935.s011.pdf]

Parts	Week	Weekly rainfall amounts (mm)															
Northern	1	5	11.7	0	0	0	88.8	43	17.2	7.6	0	0	6.2	0	0	9.7	7.4
		7.4	0	0	1.2	0	0	14.3	7.1	5.3	16.2	14.5	2.4	30	0	2.8	0
		0	10	0	4.8	21	0	30	18.2	2.8	25.9	77	0	27.2	0	30.1	17.6
		0	12.1	42.4	0	14.5	25.3	22.1	3.4	0	9.4	20.1	20.2	19.8	0		
	2	4.1	12.2	0	0	0	19.1	43.7	17.9	18.3	0	0	10.3	0	0	16.4	36.8
		24.2	0	0	16.5	0	0	23	54.1	84.4	97.1	21.5	62.9	102.1	4.8	0	6
		0	0	0	0	18.1	3.2	1.8	0	12	37.9	128.3	0.9	61.4	0	12.5	5.5
		0	2.2	14	0	12.2	54	50.5	30.3	0	0.7	49.5	24.3	39.7	0		
	3	87.9	65.6	0	0	0	88.3	111.3	69.8	72.7	0	64.5	71.7	0	0	47.6	98.3
		130.8	53.9	47.9	135.2	0	0	113	133.1	121.6	163.3	150.5	144	200.3	94.2	69.7	70
		0	55.2	18	37.7	35.5	42.8	26.3	27.8	34.2	102	209.3	57	80.7	0	23.1	71.9
		49.1	22.5	98.1	66.1	43.9	18.1	100.5	83	0	70.7	169.8	42.3	22.5	0		
	4	141.8	65	0	0	0	46.1	136.2	39.6	60.3	0	25.1	58.7	0	0	32	21.5
		57.6	22.7	0	29.5	0	0	104.5	90	97.1	120.7	121.4	56.4	119	39.2	58.8	10.3
		0	25	0	0	21.6	1.6	23.2	0	40.1	108.8	245.4	11.2	93.4	0	34.4	34
		41.2	15.5	86.1	51.9	43.3	2	65.7	42.8	0	68.4	36.8	8.9	81.5	0		
Central	1	14.3	7.2	67.5	0	0	17	4.7	8.9	3.5	0	0	0	0	0	0	0
		0	0	0	0	0	19.3	0	49.4	9.3	38.5	0	0	11.6	21	17.2	20.6
		0	0	23.8	2.3	4.1	4.2	27.6	16.4	0	0	11.3	6.5	0	27.1	0	0
		0	18.6	40.6	0	21	37.2	55.2	30.3	23.3							
	2	51.1	39.3	49.1	16.5	31.5	97.6	15.4	18	8.8	0	0	0	0	0	0	0
		0	0	0	0	0	29.8	0	19.2	14.3	11.2	0	0	25.8	5.6	0	7.6
		0	0	13.2	2	40.7	16	12.5	45.6	0	0	41.7	3	0	34.4	0	0
		0	5.9	92	0	8.6	38.3	54.4	46.3	39							
	3	69.1	44.8	73.6	47.1	28	39.7	29.5	19.1	8.9	0	0	0	0	0	0	0
		0	0	0	0	0	25.9	0	12.8	7.1	12.8	0	0	8.5	18.5	7	15.6
		0	0	20.9	14	6.8	1.9	35.1	111	0	0	5.1	5.3	0	11.7	0	0
		0	8.3	94.5	2.8	3.9	23.2	22	18.5	15							
	4	1.9	5.1	1	3.8	7	3.9	9	3.1	2.7	0	0	0	0	0	0	0
		0	0	0	0	0	0	0	35.6	4.1	23.6	0	0	0	11	0	6.3
		0	0	10.3	0.4	0.8	0.1	4.1	22.3	0	0	3.6	0	0	3.7	0	0
		0	0	229.2	0	1.1	42.7	50.3	54.6	16.4							
East	1	23.4	36.1	13.4	1.5	12.6	19.2	10	4.8	15.8	36	6.5	22.4	47.1	36.8	13.6	61
		26.3	7.5	102.5	20.4	24.9	14.6	87.5	306.9	72.8	168.3	89.5	78.6	26.5			
	2	106	49.4	118.9	117.4	110.4	9.3	13.2	1.5	33.7	33.2	4.1	23.1	48.7	81.5	24.3	70.7
		30.1	34.4	154.1	45.6	119.1	42	92.9	193.6	198.9	204.9	58.1	114.5	16.8			
	3	33.5	32.9	34	70	45.3	4.9	8	3.3	14.8	4.3	8.4	5.5	45.7	189.5	10.8	78.9
		153.9	92.5	100.5	12.5	50	23.9	316.9	585.5	248.8	113.5	79.6	253	131.9			
	4	59.1	21.8	196.4	174.6	74.5	0.3	0	0	0	0	0.1	0.3	8.7	2.7	0	10
		2	0	246.1	49.9	31.2	2.6	23	19.8	5	434.9	0	20.6	0.5			
Southeast	1	19.6	0	9.8	22.6	4.6	50.6	53	27.5	37.5	129.6	6	34.2	52.7	65.3	59.8	47.5
		45.5	50	69	34.9	7.3	5	33.4	17.9	4	13	21	0	20.2	6.8	0	32.9
		20	45.8	16.1	20.7	0	31.2	71.5	13.4	2.5	0	12.4	39.8	27.6	21.8	18	1
		0.5	0	8	20	1	10	12.8	0	4.7	0	0	70.9	26.5	12.6	35	6.8
		3.9	30.9	0.5	15.1	35	0	8	9.3	26.5	0	23.5	0	46.1	25.6	61.3	13.4
		52	86.5	21.6	3.2	25.5	19.5	15.2	0	68.6							
	2	23.5	0	34.7	2.9	12.3	115.3	116.8	182.5	92.6	319.9	155	123	240	52.3	40.3	10
		24.7	0.9	98.5	10.9	12.1	4.8	21.3	5	0	14	4	19.5	54.2	22.8	0	11.6
		72	27.4	18.2	20.5	0.5	47.2	30.4	6.2	3.3	0	13.7	0.4	13.6	4.8	0	0
		0	16	0	0	0	0	0	0	0	5.2	0	0	0	0	0	0
		0	0	0	0	0	0	0	0	0	0	0.5	0	0	0	0	0
		6.9	3.3	0	0	0	0	0	0	0							
	3	21.8	0	60.8	33.7	34	67.2	88.2	53.2	75	233.4	76.1	50.4	132	25.8	24.9	12.8
		74	0	20	22.2	12.3	40.2	29.9	6.2	9	16.2	0	25.5	63.5	22.9	2	30.4
		30	37.2	29.8	31.6	18.7	50.9	43.9	10.2	24.3	0	26.8	15.1	25.8	17.8	5.3	0
		9	40.7	9	0	0	8	22.1	25	5	0	0	7	12.1	7.5	11.3	7.5
		5.1	4.7	6.4	0.5	1.5	3.1	0	0	6.2	6.4	0	0	14.4	0	2.7	1.5
		0	17.7	0	0	4.5	0	7.1	0	0							
	4	3.4	0	13	0.5	1.6	26.2	22	59.8	11	232.4	18.3	27.1	82.5	7.1	0.2	1.9
		34.6	0	88	1.7	1.7	0.5	21	18.5	0	0	0	0	30	3.5	0	0
		25	55.5	9.3	23	0	66.7	40.2	6.9	5.5	0	0.3	15.4	3.4	30.8	0	6
		62.3	21.2	0	0	0	0	3.9	3	0	0	0	31	146.5	45.4	25.5	67.3
		34.6	65.7	107.6	11	89.6	32.6	0	116.3	104.2	59.4	0	0	47.7	85.2	250.7	112.4
		67.6	198.8	85.2	83.4	117.6	46.5	120.9	0	111.9							
Southwest	1	234.5	0	0	0	154.7	0	0	0	0	45	57.1	18.6	0	17.5	0	0
		83.5	0	0	0	0	0	0	0	0	0	0	78.6	0	29.5		
	2	334.3	0	0	0	94.8	0	0	0	0	26	52.5	34	0	27.7	0	0
		95.5	0	0	0	0	0	0	0	0	0	0	102.8	0	74		
	3	147.7	0	0	0	139.9	0	0	0	0	44.8	26.5	0	0	34.6	0	0
		103	0	0	0	0	0	0	0	0	0	0	65.8	26.3	45.9		
	4	262.9	0	0	0	43	0	0	0	0	12.6	6.3	0	0	56.2	0	0
		37.5	0	0	0	0	0	0	0	0	0	0	131.9	0	53.7		

Source: Thailand Meteorological Department. URL: https://www.tmd.go.th/services/weekly_report.php
