# Supplementary material for: Simultaneous confidence intervals for all pairwise comparisons of the means of delta-lognormal distributions with application to rainfall data
Source: PLoS One. 2021 Jul 6;16(7):e0253935. doi: 10.1371/journal.pone.0253935 (PMC8260007; doi:10.1371/journal.pone.0253935)
Supplement: S4 Table — (PDF) [file pone.0253935.s012.pdf]

Distribution	AIC values				
	Northern	Central	Eastern	Southeastern	Southwestern
Cauchy	1859.046	1131.425	1233.443	2568.220	393.113
Logistic	1831.410	1144.392	1266.594	2636.477	397.227
Lognormal	**1753.656**	**1057.969**	**1151.703**	**2401.707**	**374.674**
Normal	1844.458	1195.499	1300.466	2724.489	403.431
T-distribution	1830.311	1121.858	1234.366	2559.868	393.281
