# Supplementary material for: Simultaneous confidence intervals for all pairwise comparisons of the means of delta-lognormal distributions with application to rainfall data
Source: PLoS One. 2021 Jul 6;16(7):e0253935. doi: 10.1371/journal.pone.0253935 (PMC8260007; doi:10.1371/journal.pone.0253935)
Supplement: S5 Table — (PDF) [file pone.0253935.s013.pdf]

Estimates	Thailand parts				
	Northern	Central	Eastern	Southeastern	Southwestern
n	248	228	116	356	120
$\hat{w}$	3.40	2.61	3.38	2.89	4.03
s^2	1.37	1.46	2.56	1.91	0.76
$\hat{\delta}$	0.29	0.45	0.06	0.27	0.71
$\hat{\theta}$	41.81	15.35	96.71	33.67	23.60
