# Supplementary material for: Simultaneous confidence intervals for all pairwise comparisons of the means of delta-lognormal distributions with application to rainfall data
Source: PLoS One. 2021 Jul 6;16(7):e0253935. doi: 10.1371/journal.pone.0253935 (PMC8260007; doi:10.1371/journal.pone.0253935)
Supplement: S6 Table — (PDF) [file pone.0253935.s014.pdf]

S6 Table. 95%SCIs for all differences of weekly rainfall means.

Comparison	Mean difference	PB			FGCI			MOVER			BCI-M			BCI-U		
		L	U	Length	L	U	Length	L	U	Length	L	U	Length	L	U	Length
Northern/Central	26.46	10.82	42.09	31.27	9.12	43.80	34.68	16.10	39.44	**23.35**	-38.97	91.88	130.85	-41.68	90.22	131.90
Northern/Eastern	-54.90	-120.48	10.67	131.15	-127.62	17.82	145.44	-126.13	-21.34	**104.80**	-120.33	10.52	130.85	-120.85	11.04	131.90
Northern/Southeastern	8.14	-10.20	26.48	36.68	-12.20	28.48	40.68	-5.12	22.30	**27.42**	-57.29	73.56	130.85	-57.81	74.09	131.90
Northern/Southwestern	18.21	-2.32	38.73	41.05	-4.55	40.97	45.52	1.24	33.12	**31.88**	-47.22	83.63	130.85	-47.74	84.16	131.90
Central/Eastern	-81.36	-145.73	-16.99	128.74	-152.74	-9.98	142.76	-152.20	-49.58	**102.62**	-146.79	-15.94	130.85	-147.31	-15.41	131.90
Central/Southeastern	-18.32	-31.72	-4.92	26.80	-33.18	-3.46	29.72	-29.28	-9.18	**20.11**	-83.74	47.11	130.85	-84.27	47.63	131.90
Central/Southwestern	-8.25	-24.51	8.02	32.53	-26.28	9.79	36.07	-23.49	2.02	**25.51**	-73.67	57.18	130.85	-74.20	57.70	131.90
Eastern/Southeastern	63.04	-2.04	128.12	130.16	-9.13	135.21	144.34	30.20	134.11	**103.90**	-2.38	128.47	130.85	-2.91	128.99	131.90
Eastern/Southwestern	73.11	7.38	138.84	131.46	0.22	146.00	145.78	38.61	144.33	**105.72**	7.69	138.54	130.85	7.16	139.06	131.90
Southeastern/Southwestern	10.07	-8.81	28.95	37.76	-10.86	31.01	41.87	-6.19	23.26	**29.44**	-55.35	75.50	130.85	-55.88	76.02	131.90
