# Supplementary material for: Simultaneous confidence intervals for all pairwise comparisons of the means of delta-lognormal distributions with application to rainfall data
Source: PLoS One. 2021 Jul 6;16(7):e0253935. doi: 10.1371/journal.pone.0253935 (PMC8260007; doi:10.1371/journal.pone.0253935)
Supplement: Attachment — Submitted filename: Revisione___Simultaneous_confidence_intervals_for_all_pairwise_comparisons.pdf [file pone.0253935.s015.pdf]

Review Manuscript - PONE-D-20-38684 Simultaneous confidence intervals for all pairwise comparisons of the means of delta-lognormal distributions with application to rainfall data

This paper builds upon several works on simultaneous confidence intervals (SCI) based on parametric bootstrap, fiducial confidence interval, variance estimates recovery and credible Bayesian intervals. The latter based on mixed and uniform priors. The authors focus on the delta-lognormal distribution and motivate their study by claiming that SCI can help decision-makers to avoid the impact of unexpected events during the rainy season.

The problem of simultaneous confidence intervals is interesting, and extreme events are both challenging and of extreme importance. However, I don't think the simultaneous confidence interval for delta log-normal would help the decision-makers avoid unexpected events during the rainy season. Moreover, this paper is hard to follow due to some involving notation, that the authors should simplify. The results look simple extension of the following papers [2], [1], [3] and [4]. The authors must clarify their contribution. For example, it is not clear whether the authors propose the theorems presented in the paper. The authors must review the English. Finally, I have reported some of the main concerns.

Materials and methods.

Line 57, The authors let $Y = \log W = (Y_{j1}, \ldots, Y_{jn_{j(1)}}) \sim N(\mu, \sigma^2)$. However, Y is a vector random variable so if one assume independence than one can assume $Y \sim N(\mu, \sigma^2 I)$, where I the identity matrix of dimension $n_{j(1)}$.

Formula (3), what do the authors mean by $Var(\theta_j)$? Unless you assume a Bayesian framework and so θ_j is a random variable this doesn't make sense. Perhaps the authors intend $Var(\hat{\theta}_j)$? A similar mistake is repeated in lines 72-73. Even if the authors write $Var(\hat{\theta}_{jl})$ the equality would not hold unless you assume $Cov(\hat{\theta}_j, \hat{\theta}_l) = 0$.

Formula (6) is an approximation of the estimated variance and not equality as reported.

Formulae (7) and (8) are not correct.

Formula (13) would imply no difference between a simultaneous confidence interval derived for two or more than two groups. The authors should clarify this point. This fact also seems true for the MOVER interval.

I don't understand whether Theorem (1) is proved for $w_j > 0$, which would exclude the delta part of the log-normal distribution.

Formula (22). This quantity was introduced in formula (9). The authors call the same quantity in several ways.

Formula (28). Here the authors introduce the logarithm of the difference between two means. Then, in formula (29) they work on the difference between the means. Please be consistent.

Simulation Study

The simulation study should involve the following measures

- Coverage probability (CP),
- Upper error and lower error,
- Relative length (RL),

as defined for example in [2]. The authors considered the CP and RL. However, the definition of these two measures is not the standard one (Lines 276-278).

An Example using Real Data

The dataset seems not respect one important assumption, i.e. independence between observation within the group.

References

[1] Hasan, Md Sazib and Krishnamoorthy, K (2018). Confidence intervals for the mean and a percentile based on zero-inflated lognormal data. Journal of Statistical Computation and Simulation, 88(8), 1499–1514.

[2] Li, Xinmin and Zhou, Xiaohua and Tian, Lili (2013). Interval estimation for the mean of lognormal data with excess zeros. Statistics & Probability Letters, 83(11), 2447–2453.

[3] Donner, Allan and Zou, Guang Yong (2011). Estimating simultaneous confidence intervals for multiple contrasts of proportions by the method of variance estimates recovery, Statistics in Biopharmaceutical Research, 3(2), 320–335.

[4] Harvey, J and Van der Merwe, AJ (2012). Bayesian confidence intervals for means and variances of lognormal and bivariate lognormal distributions. Journal of Statistical Planning and Inference, 142(6), 1294–1309.
