# Supplementary material for: Simultaneous confidence intervals for all pairwise comparisons of the means of delta-lognormal distributions with application to rainfall data
Source: PLoS One. 2021 Jul 6;16(7):e0253935. doi: 10.1371/journal.pone.0253935 (PMC8260007; doi:10.1371/journal.pone.0253935)
Supplement: Attachment — Submitted filename: Response to Reviewers.pdf [file pone.0253935.s017.pdf]

Response to Reviewers

Journal: PLOS One
Manuscript ID: PONE-D-20-38684R1
Title name: Simultaneous confidence intervals for all pairwise comparisons of the means of delta-lognormal distributions with application to rainfall data
Authors: Patcharee Maneerat, Sa-Aat Niwitpong and Suparat Niwitpong

Dear Reviewers:

We would like to thank you for your time as well as patience to go over manuscript and making constructive suggestions to improve it for a resubmission. To address the specific points raised by the reviewers, the following responses are to be noted:

Reviewer #2:

1. I don't think it is necessary that each formula has its own number, I think it is enough to give a number to the ones which you further use or refer to. This makes the manuscript even easier to read. However, that's an editorial question and therefore it's not my decision if that should be changed or not.
Response: We thank the reviewer for these helpful suggestions. We agree with your comments and so have given an equation number that was referred in this revised manuscript especially.

2. Page 11, first sentence: Please delete "using RStudio". Instead, please add another line at the beginning of the results section: "R, version (...edit version...) was used for the computations of the simulations and the application." or something like that. Please note that it is not important if you use RStudio or not, it's the R version that counts.
Response: Thank you for pointing this out. This point was moved to the beginning of the results section.

3. Page 11, Simulation results, line 263: Delete "even".
Response: Line 276, it was deleted.

4. Page 16, lines 288 - 290: This sentence is grammatically a bit weird and I didn't fully understand it. Please rewrite.
Response: Thank you for pointing this out. We have rewritten in lines 301-304.

5. Page 17, line 291: Delete "the".
Response: Line 305, it was deleted.

Reviewer #3:

I am sorry, but I don't think the authors have adequately addressed my comments, and I feel that this manuscript is not acceptable for publication. Next, I will give some examples.

1. Example 1) I had raised the following point:
- The results look like a simple extension of the following papers [2], [1], [3] and [4]. The authors must clarify their contribution. For example, it is not clear whether the authors propose the theorems presented in the paper.

Authors response:
This is an excellent point raised by the reviewer. In Paragraph 4 of the Introduction, we have clarified our contribution and clarified its context in relation to studies [1–4]. In Supporting information (S1 Appendix), we have carefully checked the contexts of the theorems, as the reviewer requested.

Response: Paragraph 3 of the Introduction, we have clarified in relation to studies [1–4]. Motivated by studies [1–4] and [5-8], the proofs for the PB, FGCI and MOVER-based SCI with the asymptotic coverage property are modified for the present study, as mentioned after Eqs. (5), (10) and (13), respectively.

References:

[1] Hasan, Md Sazib and Krishnamoorthy, K (2018). Confidence intervals for the mean and a percentile based on zero-inflated lognormal data. Journal of Statistical Computation and Simulation, 88(8), 1499-1514.

[2] Li, Xinmin and Zhou, Xiaohua and Tian, Lili (2013). Interval estimation for the mean of lognormal data with excess zeros. Statistics & Probability Letters, 83(11), 2447-2453.

[3] Donner, Allan and Zou, Guang Yong (2011). Estimating simultaneous confidence intervals for multiple contrasts of proportions by the method of variance estimates recovery, Statistics in Biopharmaceutical Research, 3(2), 320-335.

[4] Harvey, J and Van der Merwe, AJ (2012). Bayesian confidence intervals for means and variances of lognormal and bivariate lognormal distributions. Journal of Statistical Planning and Inference, 142(6), 1294-1309.

[5] Hannig, J., Abdel-Karim, L. E, A. and Iyer, H. K. (2006) Simultaneous Fiducial Generalized Confidence Intervals for Ratios of Means of Lognormal Distributions, Austrian Journal of Statistics, 35(2-3), 261-269.

[6] Kharrati-Kopaei M, Eftekhar S. (2017). Simultaneous Confidence Intervals for Comparing Several Inverse Gaussian Means under Heteroscedasticity. Journal of Statistical Computation and Simulation, 87(4), 777-790.

[7] Li J, Song W, Shi J. (2015). Parametric Bootstrap Simultaneous Confidence Intervals for Differences of Means from Several Two-Parameter Exponential Distributions. Statistics & Probability Letters, 106, 39-45.

[8] Thangjai W, Niwitpong S. (2020). Simultaneous Confidence Intervals for All Differences of Coefficients of Variation of Two-Parameter Exponential Distributions. Thailand Statistician, 18(2), 135-149.

2. I checked Paragraph 4 of the Introduction, where the authors write:
"Our contribution to the field is constructing SCIs based on our proposed methods to elucidate the pairwise differences between the means of multiple delta-lognormal distributions."
which I don't think can be considered a satisfactory explanation.
Response: Paragraph 4 of the Introduction, we have revised our contribution as well as the motivation behind this study which is the estimated pairwise differences between the mean of natural rainfall records from the five areas of Thailand.

3. Example 2) Technical mistakes. I had raised the following point:
- Formula (3), what do the authors mean by Var(θ_i)? Unless you assume a Bayesian framework and so θj is a random variable, this doesn't make sense. Perhaps the authors intend Var(θˆ_j)? A similar mistake is repeated in lines 72-73. Even if the authors write Var(θˆ_ij) the equality would not hold unless you assume 〖COV(θ〗 _i,θ_j)=0.
Authors response:
We thank the reviewer for these helpful suggestions. In Eq (3), we intended using Var(θˆ_j), and so have done so throughout this manuscript. Moreover, in lines 86- 88, we assume that θˆ_j and θˆ_l are independent random variables, and so the covariance becomes 〖COV(θ〗 _i,θ_j)=0.
So, the authors correct the first technical mistake. However, it doesn't make sense to assume θˆ_j and θˆ_l as independent random variables since these quantities are estimated from the data, and so the dependence must be proved.
Response: Lines 88-92, this point has been clarified the reason of the covariance becoming $COV(\hat{\theta}_i, \hat{\theta}_j) = 0$, as shown in Crow and Shimizu [21].

Reference:
[21] Crow EL, Shimizu K. Lognormal Distributions: Theory and Applications. New York, N.Y.; Basel: CRC Press; 1988.

4. Example 3) Technical mistakes. I had raised the following point:
-Formulae (7) and (8) are not correct.
Response: Eqs (7) and (8) have been edited, and we have added the details are related to both of equations, as shown in Lines 98-99. Unfortunately, Formulae (7) and (8) are still not correct.
Response: For the PB interval, the sizes of positive observations are not equal for each resampling with replacement under bootstrap approach, so the Eqs (2) and (3) have been edited.

We look forward to hearing from you in the near future.

Sincerely yours,
Patcharee Maneerat, Sa-Aat Niwitpong and Suparat Niwitpong
The authors
