# Supplementary material for: Simultaneous confidence intervals for all pairwise comparisons of the means of delta-lognormal distributions with application to rainfall data
Source: PLoS One. 2021 Jul 6;16(7):e0253935. doi: 10.1371/journal.pone.0253935 (PMC8260007; doi:10.1371/journal.pone.0253935)
Supplement: Attachment — Submitted filename: Response to Academic Editor and Reviewers.docx [file pone.0253935.s016.docx]

**Response to Academic Editor and Reviewers**

**Journal:**  PLOS One

**Manuscript ID:** PONE-D-20-38684

**Title name:** Simultaneous confidence intervals for all pairwise comparisons of the means of delta-lognormal distributions with application to rainfall data

**Authors:** Patcharee Maneerat, Sa-Aat Niwitpong and Suparat Niwitpong

**Response to Academic Editor**

Dear Academic Editor:

We would like to thank you for your time in reviewing our manuscript. The following are our responses to each of your comments.

**Academic Editor:**

**Journal requirements:**

1. Please ensure that your manuscript meets PLOS ONE's style requirements, including those for file naming. The PLOS ONE style templates can be found at

https://journals.plos.org/plosone/s/file?id=wjVg/PLOSOne_formatting_sample_main_body.pdf

https://journals.plos.org/plosone/s/file?id=ba62/PLOSOne_formatting_sample_title_authors_affiliations.pdf

**Response:** Thank you for your suggestions. We have subsequently formatted our manuscript to follow the PLOS ONE style requirements.

2. Please ensure that you refer to Figures 1-4 in your text as, if accepted, production will need this reference to link the reader to the figures.

**Response:** We have ensured that Figures 1-4 are cited in the text of our manuscript.

3. Please upload a copy of Figures 1-4, to which you refer in your text on page 22. If the figure is no longer to be included as part of the submission please remove all reference to it within the text.

**Response:** Figures 1-4 have been uploaded with our manuscript.

4. We note you have included a table to which you do not refer in the text of your manuscript. Please ensure that you refer to Table 5 in your text; if accepted, production will need this reference to link the reader to the Table.

**Response:** Line 290, Table 5 has been cited in the text and included in the manuscript.

5. Please include captions for your Supporting Information files at the end of your manuscript, and update any in-text citations to match accordingly. Please see our Supporting Information guidelines for more information: http://journals.plos.org/plosone/s/supporting-information.

**Response:** We have included captions in the Supporting Information files and updated the citations accordingly.

Dear Reviewers:

We would like to thank you for your time and patience in reviewing our manuscript and making insightful comments toward improving it for resubmission. The following are our responses to each of your comments.

**Reviewers' comments:**

**Reviewer #1:**

The authors present a good study on the topic meeting all the necessary requirements of a new research. So I recommend the publication of the manuscript in its present form in PLOS ONE. However, the following correction may be made in the revised draft. In line 59, 'is' should be replaced with 'as'.

**Response:** Thanks for your valuable comments. In Line 73, we have replaced “is” with “as”.

**Reviewer #2:**

Major points:
1. Rewrite the manuscript as a "story" for non-statisticians. This includes

1.1 Move theorems and proofs in an appendix where people with a specific mathematical interest can find them.

**Response:** Thanks for this comment. We have moved the theorems and proofs to S1 Appendix.

1.2 Explain to non-statisticians why the issue of having simultaneous confidence bands is important.

**Response:** In Paragraph 4 of the Introduction, we have provided reasons for formulating simultaneous confidence intervals (SCIs) for the means of delta-lognormal distributions and illustrating their use on the natural rainfall data from five areas in Thailand.

1.3 Formulate with word what you are doing mathematically and why you are doing that
- Stress even more the practical use of your methods, especially in introduction and discussion.

**Response:** Thanks for your suggestions. We have attempted to do so in Paragraph 3 the Introduction and Paragraph 1 of the Discussion, as well as in the Methods by explaining the mathematical approaches in more detail.

1.4 You use a lot of abbreviations. This is necessary to avoid to lengthen the script, but it also makes it even harder to read. Maybe you could add a list of all abbreviations to look them up if necessary?

**Response:** Abbreviations used throughout this article have been added to Supporting Information (S1 Abbreviations).

1.5 I was surprised to see that the word "sampling" or "sampling distribution" never occurs in that paper although many methods are sampling-based. Please change that and give more explanations of the differences between the methods for non-statisticians.

**Response:** Thank you for your concerns. To the best of our knowledge, the word “sampling” and related phrases is a technical term in the field of statistics. For example, bootstrap sampling is a technique that involves drawing repeated samples (resampling with replacement) from the original data. (Efron B.and Tibshirani R. J., 1993). In Paragraph 2 in the Discussion section, we have explained the differences between the methods for non-statisticians.

*Reference:*

Efron B, Tibshirani RJ. An Introduction to the Bootstrap. Chapman & Hall /CRC; 1993.

1.6 To make your proposed methods useable in practice, you should not only explain the algorithms, but maybe provide the code or at least give information on statistical programs to use.

**Response:** Thank you for pointing this out. In Paragraph 2 of the Simulation Studies section, we have provided information on the statistical computing used to evaluate the SCI performances.

1.7 Take more time to explain the results of both the simulations and the application and their respective meanings.

**Response:** In Paragraph 2 of An Example using Real Data and Paragraph 3 of Discussion, we have carefully explained the empirical application associated with the simulation results.

2. I don't understand the relative average lengths. Why is MOVER taken as reference? What is meant with "less than 1 and the minimal value"? What is this minimal value? Would not a ratio smaller than 1 mean that the respective SCI is better (given a comparable coverage probability)? Because that would mean it's smaller than the corresponding MOVER SCI? I have probably understood something wrong, but this should be all the more a reason to explain that better.
**Response:** In Paragraph 1 of the Simulation Studies section, we have added a straightforward explanation of the relative average length (RAL) for easy understanding. A method with an RAL of less than 1 has a shorter average length (and thus performed better) than the one it is being compared against. Moreover, we have provided the reason why MOVER was chosen as the reference.

3. The authors use AIC on page 14 and in Table 4, but they don't explain how they get it. Give a thorough explanation how you provide those values and how the models are exactly calculated (apart from the different underlying distributions).

**Response:** In Paragraph 1 of An Example using Real Data, we have explained the use the AIC to investigate which distribution provides the best fit of the data.

4. Please mention which statistical program you used and if possible provide your code.

**Response:** As mentioned in Paragraph 2 in the Simulation Studies section, we used R studio for the statistical computing, and we added the R code to the Supporting Information.

Minor points:
1. Would it be possible to also provide the data set in the supplementary material in a form ready to be used? If one wanted to use the data in the current form one would have to type all values from Table 3 in a data set.

**Response:** As requested, we have provided the data including the R code file in the Supporting Information.

2. Sometimes definitions are hard to find or not there. For example, T on page 4 and R on page 6 seem to come out of nowhere.

**Response:** On page 4, “$T^{(PB)}$” stands for the parametric bootstrap variable, while “R” denotes the fiducial generalized pivotal quantity on page 5.

3. I couldn't find where the abbreviation "CLT" on page 9 is introduced. As said above, a list of all the abbreviations would be helpful.

**Response:** The abbreviation "CLT" has been defined and also included in the Supporting Information (S1 Abbreviations).

4. Delete the information on the flooded cave system from p.14. It has already been given in the introduction and is of no additional use here.

**Response:** Thanks for your suggestions. In Paragraph 1 of An Example using Real Data, it was deleted.

5. Mention what bold face means in the tables. I couldn't find that information.

**Response:** Tables 1-2, We added the meaning of bold face in table footnotes.

6. What does "MOVER satisfies the accurate criteria" (tables) mean?

**Response:** Thank you for your comment. We meant that MOVER’s performance was the best under those circumstances. However, we have removed this misleading text from the manuscript.

**Reviewer #3:**

1.This paper builds upon several works on simultaneous confidence intervals (SCI) based on parametric bootstrap, fiducial confidence interval, variance estimates recovery and credible Bayesian intervals. The latter based on mixed and uniform priors. The authors focus on the delta-lognormal distribution and motivate their study by claiming that SCI can help decision-makers to avoid the impact of unexpected events during the rainy season. The problem of simultaneous confidence intervals is interesting, and extreme events are both challenging and of extreme importance. However, I don’t think the simultaneous confidence interval for delta log-normal would help the decision-makers avoid unexpected events during the rainy season.

**Response:** Thank you for this important criticism. We think that the simultaneous confidence interval could provide important information that, in conjunction with other related information, can be used to make decisions that will help to prepare for extreme events in advance.

2. Moreover, this paper is hard to follow due to some involving notation, that the authors should simplify.

**Response:** Thank you for pointing this out. We moved all of the theorems to Supporting Information (S1 Appendix) where a reader with a specific interest in the mathematics can find them.

3. The results look simple extension of the following papers [2], [1], [3] and [4]. The authors must clarify their contribution. For example, it is not clear whether the authors propose the theorems presented in the paper.

**Response:** This is an excellent point raised by the reviewer. In Paragraph 4 of the Introduction, we have clarified our contribution and clarified its context in relation to studies [1–4]. In Supporting information (S1 Appendix), we have carefully checked the contexts of the theorems, as the reviewer requested.

*References:*

[1] Hasan, Md Sazib and Krishnamoorthy, K (2018). Confidence intervals for the mean and a percentile based on zero-inflated lognormal data. Journal of Statistical Computation and Simulation, 88(8), 1499-1514.

[2] Li, Xinmin and Zhou, Xiaohua and Tian, Lili (2013). Interval estimation for the mean of lognormal data with excess zeros. Statistics & Probability Letters, 83(11), 2447-2453.

[3] Donner, Allan and Zou, Guang Yong (2011). Estimating simultaneous confidence intervals for multiple contrasts of proportions by the method of variance estimates recovery, Statistics in Biopharmaceutical Research, 3(2), 320-335.

[4] Harvey, J and Van der Merwe, AJ (2012). Bayesian confidence intervals for means and variances of lognormal and bivariate lognormal distributions. Journal of Statistical Planning and Inference, 142(6), 1294-1309.

4. The authors must review the English.

**Response:** Thank you for your suggestion. The manuscript has been carefully checked by a native English speaker before resubmitting this revision.

***Materials and methods***

1. Line 57, the authors let $Y=\log W=(Y_{j1}, Y_{j2}\ldots,Y_{jn_{j\left( 1 \right)}})\sim N(\mu, \sigma^{2})$. However, Y is a vector random variable so if one assumes independence than one can assume $Y\sim N(\mu, \sigma^{2}I)$., where $I$ the identity matrix of dimension $n_{j\left( 1 \right)}$.

**Response:** Thank you for pointing this out. In this revised manuscript, we agree with your comments and so have changed it to $Y\sim N(\mu, \sigma^{2}I)$ in line 70.

2. Formula (3), what do the authors mean by $Var(\theta_{i})$? Unless you assume a Bayesian framework and so θj is a random variable this doesn’t make sense. Perhaps the authors intend $Var(\hat{\theta}_{j})$? A similar mistake is repeated in lines 72-73. Even if the authors write $Var(\hat{\theta}_{ij})$ the equality would not hold unless you assume ${COV(\theta}_{i},\theta_{j})=0$.

**Response:** We thank the reviewer for these helpful suggestions. In Eq (3), we intended using $Var(\hat{\theta}_{j})$, and so have done so throughout this manuscript. Moreover, in lines 86-88, we assume that $\hat{\theta}_{j}$ and $\hat{\theta}_{l}$ are independent random variables, and so the covariance becomes ${COV(\theta}_{i},\theta_{j})=0$.

3. Formula (6) is an approximation of the estimated variance and not equality as reported.

**Response:** Thank you for this constructive suggestion. Eq (6) has been edited in this revision.

4. Formulae (7) and (8) are not correct.

Response: Eqs (7) and (8) have been edited, and we have added the details are related to both of equations, as shown in Lines 98-99.

5. Formula (13) would imply no difference between a simultaneous confidence interval derived for two or more than two groups. The authors should clarify this point. This fact also seems true for the MOVER interval.
**Response:** Thank you for this excellent suggestion. In Lines 110-112, we have offered an explanation of why there is no difference between them (Eq 13).

6. I don’t understand whether Theorem (1) is proved for $w_{j}>0$, which would exclude the delta part of the log-normal distribution.
**Response:** Thank you for pointing this out. In Theorem 1 (S1 Appendix), we assume that the delta part is included in the information for $\theta_{jl}$, which is the parameter of interest in this study.

7. Formula (22). This quantity was introduced in formula (9). The authors call the same quantity in several ways.

**Response:** Thank you for this comment. In Eq (15), we used a beta distribution with an FGPQ of $\delta^{'}$, while for Eq (9), random samples are drawn from the sampling distribution of $\delta^{'}$ (a beta distribution)**.** Thus, a beta distribution is assumed in both but for different reasons.

8. Formula (28). Here the authors introduce the logarithm of the difference between two means. Then, in formula (29) they work on the difference between the means. Please be consistent.

**Response:** We thank the reviewer for this important criticism. The MOVER interval is suitable for the formulated CI for the combination of the parameters, so the delta-lognormal mean ($\theta_{j}$) is log-transformed as $\beta_{j}=\ln\theta_{j}=\ln\delta_{j}^{'}+(\mu_{j}+\sigma_{j}^{2})$ in Eq (20). After that, we can construct the MOVER interval for $\theta_{j}=exp(\beta_{j})$ in Eq (24). Finally, the MOVER-based SCI for $\theta_{jl}$ is obtained using Eq (25).

***Simulation Study***
The simulation study should involve the following measures

• Coverage probability (CP),
• Upper error and lower error,
• Relative length (RL),

as defined for example in [2]. The authors considered the CP and RL. However, the definition of these two measures is not the standard one (Lines 276-278).

**Response:** We thank the reviewer for pointing this out. The lower and upper error probabilities were added as measures for the SCI performances in the Simulation studies, as can be seen in Tables 1- 2.

*Reference:*

[2] Li, Xinmin and Zhou, Xiaohua and Tian, Lili (2013). Interval estimation for the mean of lognormal data with excess zeros. Statistics & Probability Letters, 83(11), 2447-2453.

***An Example using Real Data***
The dataset seems not respect one important assumption, i.e. independence between observation within the group

**Response:** We would like to thank the reviewer for pointing this important aspect out. Comparing the natural rainfall datasets for the five areas of Thailand is of interest in this study. It is possible that the observed rainfall datasets for the areas are independent due to their different climate patterns and meteorological conditions. Thailand is further sub-divided into the provinces, and there are meteorological stations in each of them. For example, in the northern area, the Chiang Mai and Uttaradit stations are located in topographical features that are conspicuously different: Chiang Mai is a city in a mountainous area while Uttaradit is in the Nan River valley with the Queen Sirikit Dam toward the north of the city. Thus, the observed rainfall amounts within the areas are independent as well.

We look forward to hearing from you in the near future.

Sincerely yours,

Patcharee Maneerat, Sa-Aat Niwitpong, and Suparat Niwitpong

The authors
